# Analysis of promoter regions of co-expressed genes identified by microarray analysis

**DOI:** 10.1186/1471-2105-7-384

**Published:** 2006-08-17

**Authors:** Srinivas Veerla, Mattias Höglund

**Affiliations:** 1 Department of Clinical Genetics, Lund University Hospital, SE-22185 Lund, Sweden

## Abstract

**Background:**

The use of global gene expression profiling to identify sets of genes with similar expression patterns is rapidly becoming a widespread approach for understanding biological processes. A logical and systematic approach to study co-expressed genes is to analyze their promoter sequences to identify transcription factors that may be involved in establishing specific profiles and that may be experimentally investigated.

**Results:**

We introduce promoter clustering i.e. grouping of promoters with respect to their high scoring motif content, and show that this approach greatly enhances the identification of common and significant transcription factor binding sites (TFBS) in co-expressed genes. We apply this method to two different dataset, one consisting of micro array data from 108 leukemias (AMLs) and a second from a time series experiment, and show that biologically relevant promoter patterns may be obtained using phylogenetic foot-printing methodology. In addition, we also found that 15% of the analyzed promoter regions contained transcription factors start sites for additional genes transcribed in the opposite direction.

**Conclusion:**

Promoter clustering based on global promoter features greatly improve the identification of shared TFBS in co-expressed genes. We believe that the outlined approach may be a useful first step to identify transcription factors that contribute to specific features of gene expression profiles.

## Background

The use of global gene expression profiling to identify sets of genes with similar expression patterns is rapidly becoming a widespread approach for understanding biological processes. Typically, gene expression data obtained by microarray analysis is organized in coherent groups of genes by several statistical means such as hierarchical clustering, self-organizing maps, *K*-means clustering, or principle component analysis. Most of these approaches readily identify clusters of tens to hundreds of genes that demonstrate similar expression patterns. Large clusters of co-expressed are frequently described as profiles or "signatures of expression" [1] that may characterize specific disease states or subtypes of e.g., tumors. Similar expression profiles may be seen in tumors of different origins suggesting co-ordination of expression at some common level. Hence, one logical systematic approach to study co-expressed genes is to analyze their promoter sequences to identify transcription factors that may be crucial for their coordinated regulation.

In eukaryotes the binding of transcription factors (TFs) to the promoter sequences results in the formation of protein complexes involving several protein-DNA and protein-protein interactions. The DNA-binding TFs recognize short DNA sequences, transcription factor binding sites, identified by various experimental methods [2-4]. By aligning sets of alternative binding sites it has been possible to determine the base pair preferences for each position within the binding site. Matrices of preferences can be transformed into a set of relative weights for each base pair in a given position. These positional weight matrices (PWM) are directly related to the relative binding energy of the protein-DNA interaction. There exist several collections of PWMs of which the most comprehensive are TRANSFAC [5] and Jaspar [6]. The PWMs provides a description of the transcription factor binding sites (TFBS) and can be used to scan genomic sequences to reveal appropriate alignments to the matrix and hence predict the location of putative TFBS. Several software tools such as MotifScanner [7], MATCH [8] and MatInspector [9] are available for these purposes. A general problem in defining TFBS is their short sequences, which make them highly abundant. Several metods have been proposed to identify binding sites showing "significance" at some level. In most cases these approaches are based on probabilities of finding the motif in a certain segment of the genome given a reference sequence [7,10,11].

In the present investigation we explore the possibility to find common TFBS patterns in promoter sequences of co-expressed genes as determined by micro array analysis. We apply two approaches to characterize promoter sequences. One that uses an analytical approach in which a given segment (600 bp) of the upstream region is searched for possible TFBS and significant binding sites are determined by a statistical scoring system based on a synthetic reference sequence [7]. A second approach is based on identifying TFBS in evolutionarily conserved sequences extracted from 3 kb upstream and approximately 1 kb downstream of the transcriptional start site [12]. In both approaches only TFBS that are significantly enriched in the promoters of the gene clusters are considered. As a final evaluation of the results we map the respective TFBS precisely to the promoter regions. We show that clustering of promoters based on motif similarity greatly enhances the identification of common TFBS patterns in co-expressed genes.

## Results

We used the QTC clustering algorithm to identify co-expressed genes in a dataset consisting of 108 acute myeloid leukemias (AML) [13]. In total 13 groups of co-expressed genes were identified using a d-value of 0.30 and with a minimum of 15 cluster members (see Additional file 1 for genes in individual gene clusters). We used Refseqs or the most 5' end of mRNAs to define the transcription start sites (TSS) and two methods to determine overrepresented transcription factor binding sites in the promoter regions. In the first approach 600 bp upstream and 100 bp downstream of the tentative TSS's were selected for analysis and significant TFBS identified using the Motifscanner software and a third-order Markov model as a background model [14]. The statistically over-represented motifs in the gene clusters were then determined as described in Aerts et al. [7]. In the second approach we selected syntenic regions between mouse and humans from 3 kb upstream and 1 kb downstream of the TSS. Evolutionarily conserved and high scoring binding sites identified by MATCH were kept for further analysis. Statistically over-represented motifs were then determined by comparing obtained results with a set of reference genes using a Mann-Whitney U-test [12]. For both approaches we added the criteria that a given binding site should be present in all promoters in a given cluster of genes to be considered significant. The first approach detected enriched binding sites in 3 out of the 13 gene clusters but in no case was these binding sites present in all promoters (Additional file 2). The second approach detected enriched binding sites in 11 of the 13 clusters, but in no case was a binding site present in all promoters (Additional file 2). Hence, no significant binding sites using the present criteria could be identified.

### Promoter clustering

**Figure 1:**
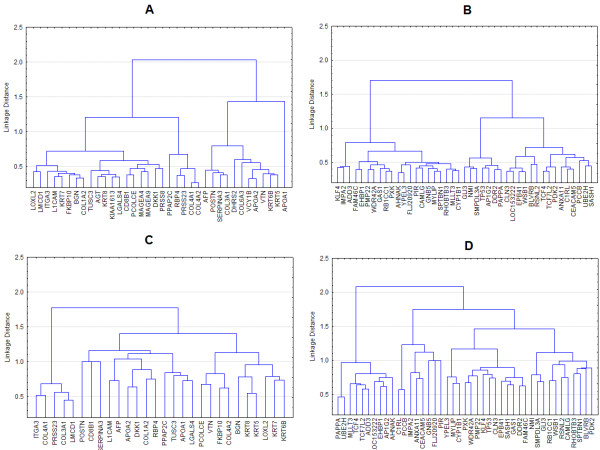
**Promoter Clustering**. [A] Promoter clustering of QTC Cluster 2 from the AML data set and [B] of QTC Cluster 7 from the serum response data set. Promoter clustering was based on TFBS present in the -600/+100 region. [C] Promoter clustering of QTC Cluster 2 from the AML data set and [D] of QTC Cluster 7 from the serum response data set using evolutionarily conserved TFBS. Genes with known orthologs are considered for the analysis.

Similar expression profiles may be caused by the coordinated action of more than one set of transcription factors. In this situation co-expression as determined by correlated expression profiles would be found but the promoter regions would be heterogeneous with respect to motif content. As a consequence, any over-representation of binding sites in a sub-set of genes would go undetected due to the presence of more than one regulated set of genes. We hypothesized that co-regulated genes would show similar or overlapping patterns of binding site motifs. To identify co-expressed genes with similar promoter organizations, each promoter sequence in a given cluster of co-expressed genes was transformed into a string of motifs based on all high scoring motifs present in the assumed promoter. To determine the similarity between promoters we used the Jaccard algorithm. This algorithm calculates similarity by estimating the fraction of shared binding sites among binding sites present in either of two promoters, and hence the algorithm does not consider the absence of a motif in two promoters as an indication of similarity. To identify subsets of genes with similar promoter regions we used hierarchical cluster analysis and the Wards algorithm for cluster formation. The hierarchical clustering generally produced two or more distinct groups of promoters in each original gene cluster, irrespectively if the -600 bp/+100 bp or the syntenic regions were used for the analysis (Figure 1). Consequently, most gene clusters were composed of genes with divergent promoters as determined by motif patterns. The resulting clusters of promoters (genes) were, however, dependent on the method used to extract the TFBS motif patterns (Figure 1). In some occasions single genes (promoters) behaved as outliers by forming a one-member subcluster.

**Figure 2:**
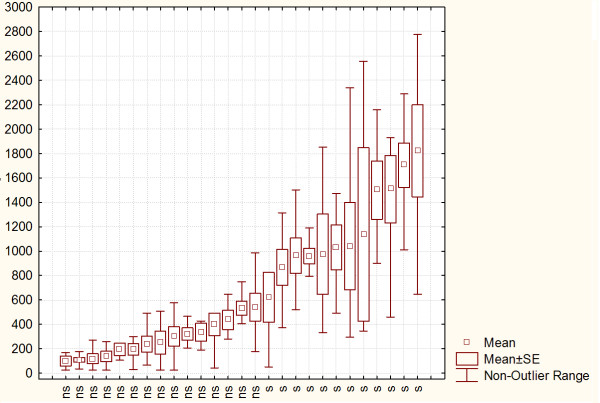
**Mean size of evolutionarily conserved regions in the gene clusters**. The clusters with non-significant binding sites (ns) have mean sizes of evolutionary conserved regions less than 600 bp and clusters with significant binding sites (s) mean sizes greater than 600 bp.

The analyses of gene promoters clustered by the -600 bp/+100 bp regions with the Motifscanner software did not yield any groups of genes with enriched binding sites present in all promoters. These results were independent of the prior probabilities and background models used [7]. The analysis of genes that showed clustering due to similarities within syntenic regions showed overrepresented binding sites in 18 of the 26 subclusters, and in 11 subclusters at least two binding sites were present in all of the investigated promoters (Table 1). The number of TFBS present in all genes within a given cluster varied between 2 and 57. No correlation between gene cluster size and the number of shared TFBS was seen. To validate the approach we produced 30 groups of 30 randomly selected genes (Additional file 3) that were subjected to promoter clustering. This resulted in a total of 67 subclusters. Each subcluster was then subjected to promoter analysis as outlined above (Additional file 4). Only seven subclusters showed two or more significant bindings sites present in all promoters, which is significantly lower than seen among the co-expressed genes (p = 0.0005, χ^2 ^test), and indicates a false discovery rate of about 10%. Even though the promoter clustering was effective in partitioning the promoters into groups with enriched and shared TFBS, no common binding sites were found in 15 out of the 26 subclusters. By plotting the average sizes of the evolutionarily conserved regions for each gene cluster it became evident that an average size of conserved sequence exceeding 600 bp (Figure 2) is necessary to identify common patterns of TFBS applying the present criteria.

### Promoter organization

**Figure 3:**
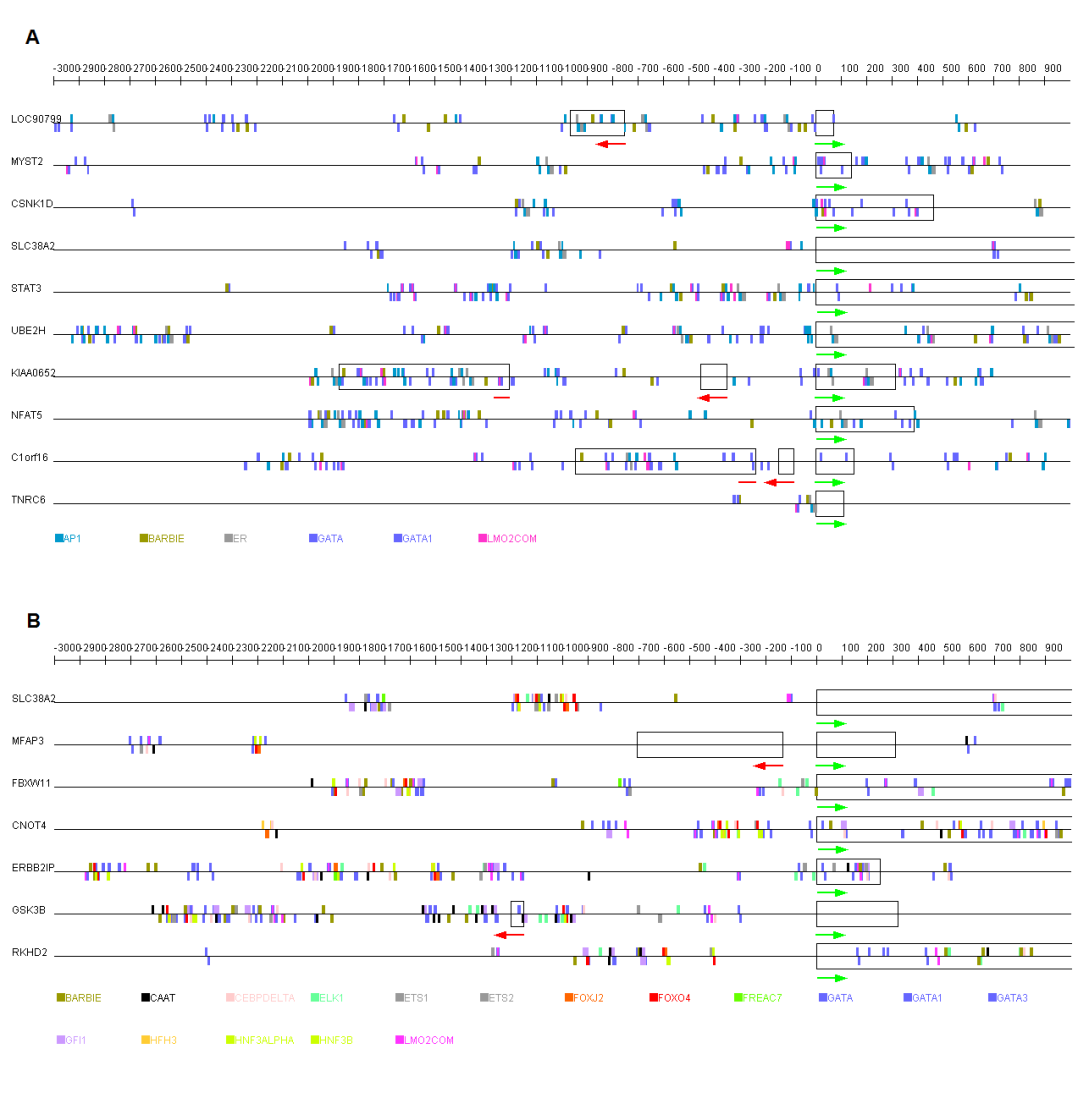
**Illustration of promoters regions obtained from analyses of the AML data set**. [A] Cluster 5C and [B] Cluster 8A. For each gene the 3 kb upstream and 1 kb downstream from the TSS is shown and the positions of significant transcription factor binding sites indicated by a color code. Boxes, exons; green arrows, transcription start sites for the investigated gene; red arrows, transcription start sites of additional genes; red bars, exons of additional genes.

To evaluate the organization of the promoters further, TFBS were mapped to the promoter sequences in the respective genes. In Figure 3 results for cluster 5C, containing 9 genes with 6 specific TFBS, and cluster 8A, containing 7 genes with 17 specific TFBS, are shown. An overall increased clustering of binding sites is seen in the proximities of the TSS. In addition, many promoters show localized clustering of several binding sites at some distance from the assumed TSS. The evolutionarily conserved TFBS in the *MFAP3 *promoter region were located in three short patches at some distance from the first exons of *MFAP3*. To investigate if the observed clustering of TFBS could be caused by alternative promoter use, each gene was checked for alterative Refseqs. No alternative promoters were however suggested. On the other hand, the 3 kb upstream regions of *LOC90799*, *KIAA0652*, and *C1orf16 *in cluster 5C contained possible TSS of additional genes. The first exon of *DDX5 *is located about 750 bp upstream of the *LOC90799 *TSS and the first exon of *FLJ32675 *is located about 300 bp upstream the *KIAA0652 *TSS. The TSS of *DKFZP564C196 *was located at position -121 bp and a second exon at position -200 bp in the *C1orf16 *promoter region. In cluster 8A the *MFAP3 *contained a TSS for an additional gene, *C5orf3*, at position -132 bp and the *GSK3B *upstream region contained the *LOC389143 *TSS at -1150 bp. In all cases was the additional gene transcribed in the opposite direction as the index gene, and hence the promoters bi-directional.

### Searching with transcription factor expression profiles

**Figure 4:**
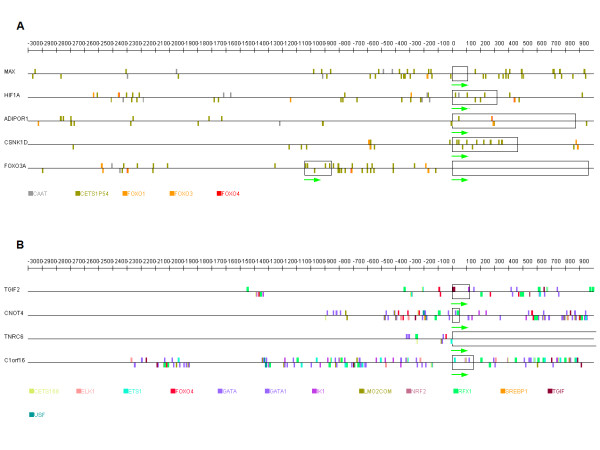
**Illustration of promoters regions obtained from analyses of the AML data set by seaching with known transcriptions factors**. [A] Cluster 1B and [B] cluster 5B. For each gene the 3 kb upstream and 1 kb downstream from the TSS is shown and the positions of significant transcription factor binding sites indicated by a color code. Boxes, exons; green arrows, transcription start sites.

We then identified all DNA binding transcription factors (DBTF) on the array that were present in the TRANSFAC database and searched for genes with expression profiles that correlated with the DBTF expression profiles. We identified 5 gene clusters with at least 15 gene members corresponding to the transcription factor genes *FOXO3A*, *MAX*, *SP3*, *STAT3*, and *TGIF2*, respectively (See Additional file 5 for the individual genes in the respective gene clusters). The evolutionarily conserved TFBS were mapped on each promoter region (-3 kb/+1 kb region) and the genes within the respective clusters subjected to promoter clustering as described above. Enriched TFBS were found in 10 of 13 subclusters, and in 6 clusters TFBS were present in all genes within the subcluster (Table 2). Cluster 1B specific for *FOXO3A *contained five genes, including *FOXO3A*, that all contained CAAT, CETS1p54, and FOXO4 sites. Furthermore, all genes contained additional FOXO1 or FOXO3A sites (Figure 4A). The organization of the *FOXO3A *upstream region showed two clusters of binding sites, and indeed two Refseqs with different 5'-ends (NM201559 and NM001455) exists for *FOXO3A *indicating the presence of alternative *FOXO3A *promoters. The fact that *FOXO3A *was a part of the clusters could indicate the presence of an auto-regulatory circuit for this gene clusters. Similarly, all genes, including *TGIF2*, within cluster 5B contained TFBS for TGIF, again suggesting an auto-regulatory loop (Figure 4B, Table 2). As TGIF2 may act as a transcriptional repressor we searched for genes negatively correlated to *TGIF2 *and performed promoter analysis. Nine out of 32 negatively correlated genes (*CCL3, DBP, ENTPD1, FOS, GLB1, NICAL, PEPD, PTPNS1*, and *UNC119*) were shown to have TGIF binding sites, ranging from 1 to 5 per promoter, we did, however, not see a significant enrichment of TGIF binding sites.

### Analyzing time series data

We then analyzed the serum induced gene expression data described by Chang et al. [15]. The co-expressed genes were identified by using the QTC algorithm and chronologically ordered with respect to appearance according to their median gene expression profiles. Syntenic regions in the -3 kb and +1 kb regions were analyzed for significant TFBS but none of the clusters showed enriched TFBS present in all promoters of their gene members. However, after promoter clustering, enriched TFBS that were present in all promoters was seen in 17 out of 43 subclusters (Table 3).

In general, TFBS for transcription factor genes expressed during the early stages of serum induction were enriched in promoters of late expressing genes. For example, *SRF *was expressed in the early gene cluster 6C and enrichment for SRF sites was seen in the late gene clusters 9A, 9C, and 2C, even though SRF binding sites was not present in all promoter sequences of the genes in these clusters. The MYC gene was expressed as a member of the gene clusters 6C and showed binding sites in all members of the simultaneously expressed clustered 6B and in 12/20 promoters in cluster 6A. MYC binding sites were also enriched in the late gene clusters 7A, with MYC sites in 5/10 promoters, and 10A, with MYC sites in 4/7 promoters, and in the late cluster 2D that showed MYC sites in all the promoters.

**Figure 5:**
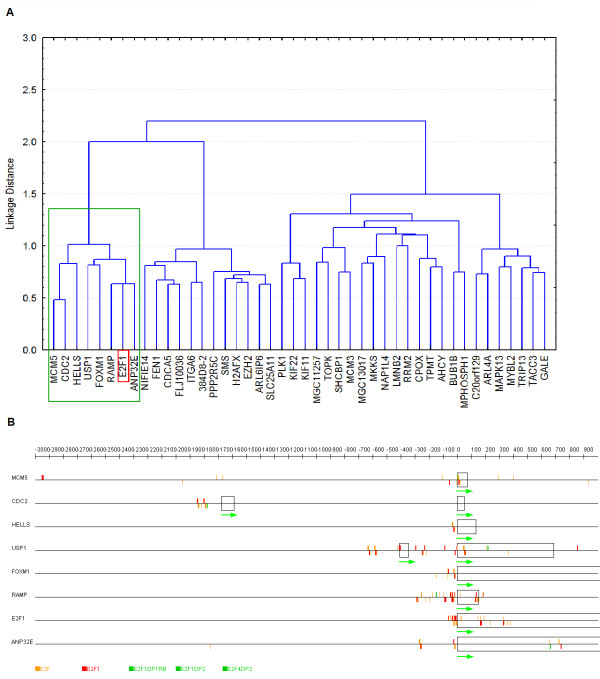
**Promoter clustering of QTC 3 genes obtained from analyses the serum response data**. [A] Subcluster 3A (green box) including the *E2F1 *gene (red box). [B] The organization of the promoters of the cluster 3A genes. For each gene the 3 kb upstream and 1 kb downstream from the TSS is shown and the positions of significant transcription factor binding sites indicated by a color code. Boxes, exons; green arrows, transcription start sites for the investigated gene; red arrows, transcription start sites of additional genes; red bars, exons of additional genes.

**Figure 6:**
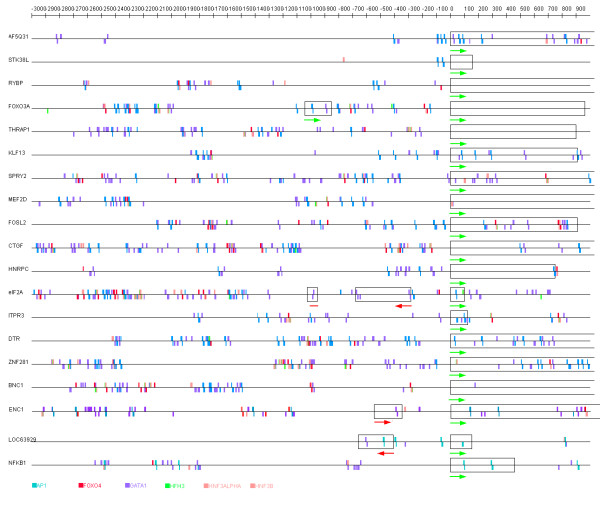
**Illustration of QTC 6A gene promoters obtained from the serum response data**. For each gene the 3 kb upstream and 1 kb downstream from the TSS is shown and the positions of significant transcription factor binding sites indicated by a color code. Boxes, exons; green arrows, transcription start sites for the investigated gene; red arrows, transcription start sites of additional genes; red bars, exons of additional genes.

Cluster 3A and 6A were selected for a more detailed analysis of the promoter organization. The late gene cluster 3A was specific for E2F1 binding sites and contained 8 genes, including *E2F1*. In addition, binding sites for E2F1DP1RB, E2F1DP2, and E2F4DP2 were present in a subset of the genes, suggesting an E2F1 auto-regulatory circuit (Figure 5). The group of TFBS identified in *CDC2 *was located about 1900 bp upstream of the indicated TSS. This TSS was determined by the start position of NM_033379. There are, however, two RefSeqs for this gene, one including (NM_001786) and one excluding (NM_033379) the first untranslated exon. When the untranslated and the first translated exons are indicated in the graph, the predicted regulatory region in *CDC2 *is located in close proximity to the untranslated first exon (Figure 5). The 6A gene cluster contained 18 genes that all showed AP1, FOXO4, GATA1, HFH3, HNF3α and HNF3β sites. Many of the promoter regions were rich in potential binding sites and the promoter regions of *MEF2D, CTGF, eiF2A, DTR, ZNF281*, and *BCN1 *showed blocks of sequences highly rich in biding sites. Two genes showed the presence of TSS for additional genes. The *eiF2A *promoter region contained the TSS for *SERP1 *336 bp up stream of the *eiF2A TSS*, and the *LOC63929 *contained the TSS of *ST13 *at position -460 bp relative the *LOC63929 *TSS. The *ENC1 *promoter region contained the TSS for *LOC401199 *at position -521 and in the same orientation as *ENC1*; in fact the non-coding first exon of *ENC1 *is the second coding exon of *LOC401199 *(Figure 6).

## Discussion

In the present investigation we have pursued the presence of common regulatory motifs in co-expressed genes identified by microarray analysis. One of our aims was to investigate the possibility to reduce gene expression data obtained by whole genome microarray analyses into hypotheses regarding transcription factors responsible for features of the expression profiles. We analyzed real microarray data in the form of two different data sets, one composed of expression profiles from 108 leukemia [13] and one of serum induced expression changes in resting fibroblasts [15]. The two datasets hence represent two different types of data, one with several stationary states i.e., different tumor cases, and one a transition from one state to another in the form of a time series experiment. The major assumption was that co-regulated genes show enrichment for common transcription binding sites in their promoter regions.

A crucial step in the analysis is to determine the transcription start sites. We made use of Refseqs or, when RefSeqs were not available, the longest available mRNA sequence to determine putative TSS. Even though this strategy will not determine the precise position of the TSS in all instances we assumed that by analyzing relatively large regions covering the tentative TSSs relevant TFBS would be detected. Sequence comparisons of human and mouse genes has revealed that homologous sequences in the vicinity of the TSS have an average length of about 510 bp [16]. Hence in a first approach we first limited the analysis to the 600 bp upstream and 100 bp downstream of the TSS. In this approach we used Motifscanner to identify high scoring TFBS in the promoter regions of the respective co-expressed genes and then identified overrepresented sites using the algorithm of Aerts et al. [7]. As an alternative approach we used phylogenetic foot-printing [17]. Phylogenetic foot-printing is based on the notion that non-coding genomic sequences important for gene regulation will be more highly conserved than segments that has no influence on gene expression. Hence, several algorithms limit the analysis to syntenic regions shared by two or more sequenced species [18]. The analysis of conserved non-coding has led to the conclusion that sequences also located to introns and to within 5 kb upstream of tentative TSSs may be of importance for gene regulation [19]. A further development is to only consider evolutionarily conserved TFBS.

To identify groups of co-expressed genes we used the QTC algorithm [20]. The QTC algorithm works by forming a candidate gene cluster with the first gene as a seed and grouping genes with the highest correlation iteratively in a way that minimizes the cluster diameter d, until no further genes may be added without exceeding a predetermined d-value. This procedure is performed with all genes in the data set as a seed. The largest cluster is then retrieved and the procedure repeated excluding the genes comprising the first cluster. This makes sure that the largest and most coherent clusters of genes are formed. An advantage of this method is that the quality of the gene clusters, the width of the cluster, may be adjusted by tuning the d-value. However, despite the fact that reasonably narrow gene clusters were used, the initial analyses revealed very few groups of co-expressed genes with enriched and common TFBS. We then reasoned that similar expression profiles might be caused by the coordinated action of more than one set of transcription factors. A biological situation in which this may be conceived is the alteration of gene expression induced by the activation of a receptor protein. In this case genes may be activated in a coordinate fashion through different paths e.g., the RAS-RAF-MEK and the JAK-STAT pathways that ultimately induces different sets of transcription factors in a coordinated fashion. An alternative situation is a hierarchical organization of TFs in which one transcription factor, or one set of transcription factors, activates sets of downstream transcription factors, which in turn induce the cellular response. In both these scenarios co-expression would be found but the promoters responsible for the final response would be activated by more than one set of transcription factors. As a consequence, an enrichment of binding sites could go undetected due to its presence in only in a sub-set of promoters. To identify co-expressed genes with similarly organized promotors we transformed each promoter into a string of significant or evolutionarily conserved TFBS present in the promoter and then calculated the Jaccard's distances between promoters. The Jaccard algorithm estimates the fraction of shared binding sites in two promoters and it does not consider a shared absence of a TFBS as an indication of similarity. This latter feature is important as there will be promoters negative for a large number of cis-elements when comparing large number of genes. An analogous measure of promoter similarity has been proposes by Hannenhalli and Levy [21].

By repeating the analyses of co-expressed genes that also showed similar promoter regions several biologically relevant promoter patterns were identified. The analysis of the serum induced gene expression revealed a temporal organization of TF expression and enrichment for the corresponding TFBS in promoters of the late expressing genes. The *MYC *gene was expressed as a member of early gene cluster 6. Interestingly, *MYC *was expressed as a part of subcluster 6C, in which no significant TFBS were identified, whereas MYCMAX sites were present in all cluster 6B genes and in 12 out of the 20 cluster 6A genes. A possible interpretation of this data is that an external signal activate *MYC *which in turn, as an immediate response, activates genes in clusters 6A and 6B which hence show coordinated expression with *MYC*. MYCMAX sites were also enriched in the later clusters 7A and 10A, and present in all of the promoters in the late gene cluster 2D. Hence, these clusters may represent a late response to MYC activation. A similar temporal organization of *SRF *expression and SRF binding sites was also suggested. A potential regulatory circuit involving E2F1 was identified in the time series data. This circuit included *MCM5 *and *CDC2*, both known to be regulated by E2F1 [23,24], as well as the lymphoid-specific helicase *HELLS *and *USP1*. *USP1 *is induced during S-phase and switches off FANCD2-mediated DNA repair as cells enter G2/M, or once DNA repair is completed, by promoting FANCD2 de-ubiquitination. Hence, both HELLS and USP1 are associated with DNA replication. The promoter regions of these genes contained several E2F and E2F related binding sites in close vicinity of their TSS. The fact that the transcription factor E2F1 is a part on the gene cluster suggests that the expression of the other genes in the cluster is maintained once E2F1 is activated. Similar auto regulatory loops were also suggested for the *FOXO3A *and *TGIF1 *transcription factors.

**Figure 7:**
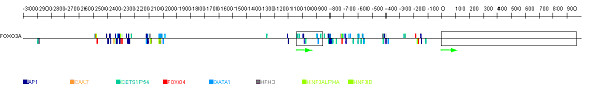
**A tentative consensus *FOXO3A *promoter organization**. The 3 kb upstream and 1 kb downstream from the TSS is shown and the positions of significant transcription factor binding sites indicated by color codes. Boxes, exons; green arrows, transcription start sites.

In at least two genes, *FOXO3A *and *CDC2*, alternative promoters were suggested. In the *CDC2 *case several E2F related TFBS preceded the most upstream first exon whereas no significant cis-elements were seen in the proximity of the alternative downstream first exon. Both the human coding sequence and the mouse ortholog *cdc2a *show an untranslated first exon. In humans two Refseqs exist for *CDC2*, one including and one excluding the untranslated first exon, that both result in the same protein whereas the mouse ortholog only produce one transcript (NC_000076). This may indicate that the second human promoter have evolved after the divergence of human and mouse and hence that this promoter is not detected by phylogenetic foot-printing. In *FOXO3A*, clusters of TFBS preceding the two alternative first exons were clearly seen. By combining information from the AML and the time series data a tentative consensus *FOXO3A *promoter organization may be obtained (Figure 7). This derived organization of the promoter region shows that the most upstream promoter contain 9 AP1, 2 CAAT, 7 CETS1P54, 3 FOXO4, 10 GATA1, and 6 HFH3 sites whereas the second promoter contains 7 AP1, 20 CETS1P54, 3 FOXO4, 10 GATA1 and 3 HFH3 sites. Hence, the major differences between the promoters are the numbers of CETS1P54 and HFH3 sites.

The investigation also identified some potential limitations of the approach. Several of the analyzed promoter regions showed the presence of TSS for additional genes. The associated genes were transcribed in the opposite direction to the index genes in all but one case and hence the promoters were bi-directional. The observed frequency, 15%, of bi-directional promoters is close to the fraction believed to be present in the human genome [22]. Bi-directional promoters may at first hand seem to complicate the analysis. However, as these promoters coordinate the expression of the two flanking genes and anti-regulation is believed to occur only in a minority of the cases [22], they may be treated as single promoters.

In spite of the stringent criteria used to identify significant binding sites, common sites were found in a large proportion of the investigated gene clusters. The limiting feature in the remaining clusters is most likely related to the average size of the conserved sequences in the promoter regions. We could show that the average size of the evolutionarily conserved regions had to exceed 600 bp to result in positive outcomes using the indicated criteria for conserved sequences and for high scoring PWM's. Hence, only clusters of co-expressed genes in which all members show considerable conserved regions within their promoter regions are amiable for analysis. The fraction of promoters amenable for analysis would most likely be increased if the criteria for identifying evolutionarily conserved segments or for identifying putative bindings sites were relaxed. A possible alternative approach would thus be to treat each cluster of promoters individually and decrease the criteria in a stepwise fashion until an optimum setting is reached for each promoter cluster. Even though there may be advantages of such a procedure, it is also linked with several computational problems, such as determining when optimal settings have been achieved. This approach was therefore not pursued further.

Even though mapping of significant binding sites within the promoter regions frequently revealed clusters of TFBS in close vicinity of the TSS, a large fraction was also located at some distance form the TSSs. A particular feature was the presence of segments 100–600 bp in size with a high density of cis-elements. A striking example of such organization is the promoter of *MFAP3 *with three short sections of binding sites at some distance from the TSS and no significant sites at the TSS. In some genes these segments coincided with exons but in most cases not. The nature of these high-density TFBS regions may be several. Some may have enhancer element activities and may rather be involved in recruiting transcription factor molecules than in the actual initiation of transcription. Alternatively, promoters may contain evolutionarily conserved regions not involved in transcription. In this latter case TFBS detected within the segments will still be evolutionarily conserved but not functional e.g., a first step to unravel the nature of these regions would be to compare the bioinformatical data with chromatin immunoprecipitation analyses [25].

## Conclusion

We have shown that that it is possible to identify biological relevant patterns in promoters of co-expressed genes using microarray data. A critical step to succeed in this analysis was not only to identify genes with correlated expression but also to classify genes with respect to global promoter features. Our intention was not to arrive at a fully working method for the analysis of promoter regions. We believe that this goal can only be reached if a large number of practical problems are solved, problems that may take several investigation to resolve. Our starting point was microarray data and the question if given a microarray data set it is possible to produce hypothesis about transcription factors that may be responsible for some of the features in the expression profiles. Even though our aim was not to map promoters in detail we believe that the outlined approach may be a useful first step to understand the underlying factors that determines specific features of gene expression profiles.

## Methods

### Data sets

The AML dataset described by Bullinger et al. [13] was downloaded from the Gene expression Omnibus [26] to contain 6283 genes/reporters. Eleven cases showed a high frequency of missing values (>1800 missing values) and were excluded from further analyses. Reporters for identical genes were merged and genes with at least 80% values were selected and corrected for missing values by KNN imputation using K = 12 [27]. The final data set included 4651 genes and 108 cases. The time series data described by Chang et al. [15] was downloaded from the Stanford Microarray Database [28]. Reporters for identical genes were merged and genes with at least 80% values were selected and corrected for missing values by KNN imputation using K = 12 resulting in a dataset of 568 genes and 16 time points. Expression values for t = 0 was obtained by the mean expression values of all experiments designated t = 0.

### Clustering methods

To find genes with similar expression we used QTC (Quality Cluster algorithm) [20] and PTM (Pavlidis Template Matching) [29]. QTC works by forming a candidate cluster of the first gene and grouping genes with the highest correlation iteratively in a way that minimizes the cluster diameter d, until no further genes may be added without exceeding a predetermined d-value. This procedure is performed with all genes in the data set as a seed. The largest cluster is then retrieved and the procedure repeated excluding the genes selected for the preceding cluster. This makes sure that the largest and most coherent clusters of genes are formed. We used diameter 0.3 and the cluster size of at least 15 members for the AML data, and diameter 0.2 and the cluster size of at least 15 members for time series data. The d-values were adjusted empirically to result in reasonably small clusters of genes, 50 or less, showing high correlation. The minimum number of cluster members was set to 15 to ensure that a sufficient number of the identified genes also showed mouse orthologs. We identified genes encoding DNA binding transcription factors using GO id GO:0003677 from the AmiGO database [30] and retrieved genes with similar expression profiles by using the PTM clustering algorithm. The PTM algorithm forms clusters by finding the correlation between two profiles. We used a Pearson correlation value r ≥ 0.6 for the AML dataset and a minimal cluster size of 15, for the time series data we used r ≥ 0.85 and a minimal cluster size of 15. We used the TMeV software [31] to perform the above clustering algorithms.

### Promoter sequences and identification of mouse orthologs

We used RefSeqs start positions [32] and for genes with no RefSeqs the most 5' mRNA sequence information as TSS [33]. We retrieved RefSeqs, mRNA information, and promoter regions from the UCSC Genome browser database [34] and downloaded orthologous mouse gene information from the from the JaxOrtholog table [35]. In cases where orthologous mouse gene information was not present in the Jaxortholog table, we use the HomoloGene position information [36] and retrieved the promoter regions manually from the UCSC. Promoter regions, 3000 bp upstream and 1000 bp downstream of the tentative TSS, for these genes were retrieved from the UCSC Genome browser database. We used the BLAST program [37] to retrieve homologous sequences and the criteria e<0.001 to identify evolutionary conserved regions.

### Mapping and computing significance of TFBS

Two different approaches for mapping of TFBS and calculating their significance were used. First the Motifscanner program, implemented in the TOUCAN software [7], was used to map the TFBS and a third-order Markov background model was used to identify significant binding sites [14]. We used a prior probability of 0.2, the probability of finding one instance of a given motif model and the background model, as this is the standard setting in the TOUCAN software [7] and analyzed 600 bp upstream and 100 bp downstream of the putative transcription start site. For the analyses of evolutionary conserved regions in promoters we used the CONFAC software [12]. This software maps TFBS using the MATCH program [8] and estimates the enrichment for specific TFBS by a Mann-Whitney U-test using random gene list as a reference. We used a core matrix score ≥ 0.95 and a matrix similarity score ≥ 0.85 to identify putative binding sites. The TRANSFAC v.8.3 database containing 243 vertebrate PWMs was used to identify transcription factor binding sites.

### Promoter clustering

To identify similar promoters each promoter sequence was first transformed into a string of TFBS motifs. Groups of promoters were then transformed to a matrix format in which columns correspond to specific promoters and rows to presence or absence of individual TFBS. Hence, if the gene with largest number of E2F binding sites has ten such sites and the gene with next largest number has eight sites there will be ten rows for E2F in which the first gene will be scored with "presence" in ten rows and the second gene with "presence" in eight and "absence" in two. In this way, not only the presence or absence of the TFBS is considered but also the number of binding sites. These matrices were then used to generate similarity matrices using Jaccards algorithm. This algorithm does not consider the absence of binding sites in two promoters as an indication of similarity. If a simple matching coefficient is used some promoters would appear very similar primarily because they both lack the same features rather than because the features they do have is shared. Hence, as the more commonly used similarity measures based on Euclidian distance or Pearson correlation would take shared absence of binding sites as a sign of similarity, these measures may produce misleading estimates. To calculate the Jaccards coefficient let A be the sum of matches (1, 1) that is the number of TFBS present in both genes G1 and G2, B and C be sum of mismatches (1, 0) and (0, 1) that is the number of TFBS present in either of G1 or G2, and D be sum of concomitant absence (0, 0) that is absence in both genes G1 and G2, then the Jaccards similarity value (S) for these two genes is S(G1,G2) = A/(A+B+C). To identify genes with similar promoters we used hierarchical cluster analysis using 1-S as a dissimilarity measure and Wards algorithm for cluster formation.

## Authors' contributions

MH conceived the investigation, SV performed all the computations, bioinformatical analyses, and programming. SV and MH contributed to drafting the manuscript.

## Supplementary Material

Additional File 1 Gene members in the AML QTC clusters. Click here for file

Additional File 2 Significant transcription factor binding sites detected in the AML QTC clusters using the TOUCAN and the CONFAC software. Click here for file

Additional File 3 Randomly selected gene groups. Click here for file

Additional File 4 Promoter analysis for the randomly selected gene groups. Click here for file

Additional File 5 Gene members in the AML PTM clusters. Click here for file

## Figures and Tables

**Table 1:** Significant TFBS of QTC subclusters from the AML data set.

**Clusters**	**Subclusters**	**Number of genes**	**Significant TFBS present in all promoters of a given cluster**
1	1A	13	Significant TFBS but not present in all
	1B	16	No significant
2	2A	7	AP1, CETS1P54, ETS1, HAND1E47, MYOD
	2B	7	No significant
	2C	14	No significant
3	3A	3	AHR, AP1, AREB6, ATATA, BARBIE, CAAT, CDPCR3HD, CEBPDELTA, CETS168, CETS1P54, CHOP, CP2, E2F1, ELK1, ETS1, ETS2, FOXD3, FOXJ2, FOXO4, FREAC2, FREAC7, GATA, GATA1, GATA2, GATA3, GFI1, HAND1E47, HEB, HFH3, HFH8, HNF3ALPHA, HNF3B, HNF6, IK1, IPF1, LDSPOLYA, LHX3, LMO2COM, MAZ, MTATA, MYOD, NFY, NKX25, OCT1, PADS, POU1F1, RFX1, S8, SOX9, TAL1BETAE47, TATA, TCF1P, TGIF, TITF1, XFD1, XFD3, ZTA
	3B	12	No significant
	3C	6	CAAT, CMYB, FOXO4
4^1^	-	-	-
5	5A	6	Significant TFBS but not present in all
	5B	3	No significant
	5C	10	AP1, BARBIE, ER, GATA, GATA1, LMO2COM
6	6A	8	Significant TFBS but not present in all
	6B	7	Significant TFBS but not present in all
7	7A	3	FOXO4, MYOD
	7B	6	No significant
8	8A	7	BARBIE, CAAT, CEBPDELTA, ELK1, ETS1, ETS2, FOXJ2, FOXO4, FREAC7, GATA, GATA1, GATA3, GFI1, HFH3, HNF3ALPHA, HNF3B, LMO2COM, TGIF
	8B	6	No significant
9	9A	7	No significant
	9B	5	CETS168, IK1, MYOD, NRF2
10	10A	4	Significant TFBS but not present in all
	10B	5	AP1, CETS1P54, ER, FOXO4, GATA1
11	11A	5	No significant
	11B	6	GATA, IK1, MYOD
12	12A	4	GATA1, GATA2
	12B	7	Significant TFBS but not present in all
13	13A	3	No significant
	13B	5	CETS168, CMYB, IK1, MYCMAX, NRF2, TFIII

^1 ^No subclusters formed.

**Table 2:** Significant TFBS of PTM subclusters from the AML data set.

**Cluster**^1^	**Subclusters**	**Number of genes**	**Significant TFBS present in all promoters of a given cluster^2^**
FOXO3A	1A	3	No significant TFBS
	1B	6	CAAT, CETS1P54, FOXO4 (FOXO1 (4/6) FOXO3 (4/6))
	1C	4	No significant TFBS
MAX	2A	12	Significant TFBS but not present in all
	2B	13	AP1, BARBIE, GATA, GATA1, HFH3, LMO2COM
SP3	3A	6	Significant TFBS but not present in all
	3B	8	No significant TFBS
	3C	5	Significant TFBS but not present in all
	3D	13	AP1, BARBIE, CAAT, CEBPDELTA, CETS1P54, FOXO4, GATA, GATA1, LMO2COM, NKX25, S8
STAT3	4A	6	MAZ, NRF2
	4B	6	AP1, GATA, GATA1, LIMO2COM
TGIF2	5A	7	Significant TFBS but not present in all
	5B	4	CETS136, ELK1, ETS1, FOXO4, GATA, GATA1, IK1, LMO2COM, NRF2, RFX1, SREBP1, TGIF, USF

^1 ^DNA binding transcription factor used for cluster formation.

^2 ^Significant TFBS present in all promoters of the subcluster genes and the TFBS in parenthesis are present in the fraction promoters indicated.

**Table 3:** Significant TFBS of QTC subclusters from the serum response data set.

**Cluster**	**Time point**	**Number of genes**	**Significant TFBS present in all promoters of a given cluster**^1^	**DBTF part of gene cluster**^2^
11A	3	8	AP1, ETS1, RFX1 (SRF 3/8)	
11B	3	12	Significant TFBS but not present in all	
1A	4	14	No significant TFBS	
1B	4	8	CEBPDELTA	
1C	4	21	Significant TFBS but not present in all	
1D	4	17	No significant TFBS	
6A	4	20	AP1, FOXO4, GATA1, HFH3, HNF3ALPHA, HNF3B (MYCMAX 12/20))	
6B	4	7	ARNT, HIF1, MYCMAX, NMYC, USF	
6C	4	24	No significant TFBS	MYC, SRF
8A	4	17	Significant TFBS but not present in all	
8B	4	10	Significant TFBS but not present in all	
4A	5	11	No significant TFBS	
4B	5	10	CETS1P54, ETS1, ETS2, IK1	
9A	5	6	AP1, BARBIE (SRF 2/6)	
9B	5	15	No significant TFBS	
9C	5	13	Significant TFBS but not present in all (SRF 3/13)	
14A	5	5	No significant TFBS	
14B	5	6	Significant TFBS but not present in all	
14C	5	10	Significant TFBS but not present in all	
12A	5	11	Significant TFBS but not present in all	
12B	5	15	RFX1	
7A	6	10	AP1, BARBIE, CETS1P54, CREL, FOXJ2, FOXO4, GATA1, HAND1E47, HFH3, HNF3ALPHA, HNF3B, IK1, NKX25, RFX1 (MYCMAX 5/10, TCF4 6/10)	TCF4
7B	6	8	No significant TFBS	
7C	6	14	FOXO4	
7D	6	11	Significant TFBS but not present in all	
15A	6	17	Significant TFBS but not present in all	
15B	6	6	No significant TFBS	
5A	7	13	AREB6, IK1	
5B	7	5	CAAT, NFY	
2A	9	19	No significant TFBS	
2B	9	16	Significant TFBS but not present in all	
2C	9	16	AP1 (SRF 4/16)	
2D	9	7	ARNT, HIF1, MYCMAX, NMYC, USF	
10A	9	7	AP1, BARBIE, CAAT, CDPCR3HD, CABPDELTA, E2F1, ETS1, FOXJ2, FOXO4, GATA, GATA1, GFI1, HFH3, HFH8, HNF3ALPHA, IPF1, LMO2COM, MTATA, MYOD, TGIF (MYCMAX 4/7, TCF4 5/7)	
10B	9	16	No significant TFBS	
13A	10	10	GATA1	
13B	10	12	No significant TFBS	
3A	12	8	E2F, E2F1 (E2F1DPIRB (7/8), E2F1DP2 (5/8) E2F4DP2 (5/8)	E2F
3B	12	12	GATA1	
16A	-^3^	7	No significant TFBS	
16B	-	18	No significant TFBS	
17A	-	8	Significant TFBS but not present in all	
17B	-	11	No significant TFBS	

^1 ^TFBS in parenthesis are present in the fraction of promoters indicated.

^2 ^DNA binding transcription factors present in the respective subclusters.

^3 ^Indicates flat profiles.
