# Supplementary material for: Analysis of promoter regions of co-expressed genes identified by microarray analysis
Source: BMC Bioinformatics. 2006 Aug 17;7:384. doi: 10.1186/1471-2105-7-384 (PMC1560170; doi:10.1186/1471-2105-7-384)
Supplement: Additional File 1 — Gene members in the AML QTC clusters. [file 1471-2105-7-384-S1.doc]

## Additional File 1. Gene members in the AML QTC clusters.

| **Cluster 1** | **Cluster 2** | **Cluster 3** | **Cluster 4** | **Cluster 5** | **Cluster** **6** | **Cluster 7** | **Cluster 8** | **Cluster 9** | **Cluster 10** | **Cluster 11** | **Cluster 12** | **Cluster 13** |
| --- | --- | --- | --- | --- | --- | --- | --- | --- | --- | --- | --- | --- |
| ASGR2  CAMK1  CCR1  CD14  CD68  CDK7  CSPG2  CST3  CYBB  CYP51A1  FCER1G  FGL2  IFI30  IL13RA1  ITGAL  ITGAM  KRT5  LOC152195  PRRG1  PTPNS1  RAD23A  RER1  S100A11  S100A4  SEMA4A  TNFRSF1B  TYROBP  VDR  ZNF451 | AFP  APOA1  APOA2  BGN  CD8B1  COL1A2  COL3A1  COL4A1  COL4A2  DKK1  FKBP10  ITGA3  KRT5  KRT6B  KRT7  KRT8  L1CAM  LGALS4  LMCD1  LOXL2  PCOLCE  POSTN  PPAP2C  PRSS23  RBP4  SERPINA3  TUSC3  VTN | ALAS2  BCL2L1  BPGM  C14orf87  CGI-69  E2-230K  EPB41  EPB49  FECH  FLJ43855  GSPT1  HAGH  HMBS  MIR  MSCP  MXI1  OSBP2  PRDX2  SELENBP1  SNCA  TM4SF9 | C10orf3  C18orf24  CDC6  CDCA8  COBL  FEN1  HSPC150  KIAA0101  LOC388962  MAD2L1  MCM3  MTHFD1  MYBL2  PRIM1  RECQL4  UBE2C | ACIN1  C1orf16  C20orf140  CSNK1D  DVL3  KIAA0652  LOC90799  MAP3K7IP2  MAX  MYST2  NFAT5  PRPF8  SLC38A2  SP3  SPTBN1  STAT3  TNRC6  UBE2H  ZNF326 | ARV1  B3GALT6  DKFZP564A022  DNCL1  EXOSC5  EXTL2  GTF2H1  HDHD2  ICMT  KIAA0179  LMAN2L  PIGN  SRD5A2L  UBE2L3  VKORC1L1 | APOBEC3B  ASAH1  BST1  CYP1B1  FLJ20273  GALNAC4S-6ST  LIG4  LILRB2  NINJ1  PIK3R5  S100A8  SLA  TNFRSF1B | API5  CNOT4  CNOT6L  ERBB2IP  FBXW11  FLJ12529  GSK3B  KIAA1109  KPNA5  MFAP3  MOBK1B  RABL3  RKHD2  SLC38A2  TMEM33  XRN1 | ARFRP1  C6orf108  CDK10  DCPS  EIF4EBP1  FBXL7  GLIS2  LOC221143  MAD1L1  Magmas  PAK4  POLR2H  RNASEH2A  SDCCAG10  SEMA5A  TST | AHNAK  C14orf43  CENTB5  CLSTN1  EP400  FLJ30656  INPPL1  JAK1  KIAA1536  MAP3K1  MGC4796  MYH11  NFIC  TGOLN2 | ACTB  ARRB2  ASGR1  CAPN2  CTSB  GSTM3  HEXB  IL13RA1  KIF1C  LCP1  MAP2K1  NELL1  RIN1  SQRDL | APOB  C11orf13  CALD1  CDH13  DSP  HSPB8  ITIH2  KRT13  KRT19  LOC51066  PCDH1  PCDHA6  TF  URB  ZD52F10 | C14orf118  C20orf108  EWSR1  HNRPD  HNRPM  LOC168850  OSBP  PDLIM2  RB1CC1  RBM5  TRA2A  YT521 |
