# Supplementary material for: Analysis of promoter regions of co-expressed genes identified by microarray analysis
Source: BMC Bioinformatics. 2006 Aug 17;7:384. doi: 10.1186/1471-2105-7-384 (PMC1560170; doi:10.1186/1471-2105-7-384)
Supplement: Additional File 2 — Significant transcription factor binding sites detected in the AML QTC clusters using the TOUCAN and the CONFAC software. [file 1471-2105-7-384-S2.doc]

**Additional File 2. Significant transcription factor binding sites detected in AML QTC clusters using the TOUCAN and the CONFAC software ^1^**

| **Cluster No.** | **Total. No. of genes.** | **No. of orthologs** | **No of genes with conserved sequences** | **Significant**  **Binding sites**  **(TOUCAN)** | **Significant binding sites** (CONFAC) |
| --- | --- | --- | --- | --- | --- |
| 1 | 46 | 42 | 29 | SREBP1_01 | E2F |
| 2 | 36 | 32 | 28 | No sig. | E2F, GATA1 |
| 3 | 25 | 25 | 21 | No sig. | NFKB, CREL, CP2, GATA1, LPOLYA, HAND1E47, AHR |
| 4 | 27 | 26 | 16 | NFY_01 | No sig. |
| 5 | 27 | 23 | 19 | No sig. | AHR,ATF,ATF4,CAAT,CREB,CREBP1,CREL,  E2F,ELK1,FOXO4,GATA1,IPF1,MAZ,NFKB,  NFY,RFX1,TFIII,USF,VMYB |
| 6 | 18 | 17 | 15 | USF, MAX | No sig. |
| 7 | 13 | 12 | 9 | No sig. | AHR |
| 8 | 16 | 14 | 13 | No sig. | AHR,E2F,ELK1,LPOLYA,NFKB,TGIF |
| 9 | 16 | 16 | 12 | No sig. | NFKB |
| 10 | 14 | 13 | 10 | No sig. | BACH1,BACH2,CETS168,CREL,GABP,  MAZ,NFKAPPAB,NFKB,NRF2,SREBP1,SRF |
| 11 | 14 | 13 | 11 | No sig. | ELK1,E2F |
| 12 | 15 | 14 | 13 | No sig. | NFKB |
| 13 | 12 | 12 | 11 | No sig. | ARNT, E2F, HIF1, HNF4, MAX, MYCMAX |

^1^No sig., no significant transcription factor binding sites.
