# Supplementary material for: Analysis of promoter regions of co-expressed genes identified by microarray analysis
Source: BMC Bioinformatics. 2006 Aug 17;7:384. doi: 10.1186/1471-2105-7-384 (PMC1560170; doi:10.1186/1471-2105-7-384)
Supplement: Additional File 3 — Randomly selected gene groups. [file 1471-2105-7-384-S3.doc]

| **R1** | **R2** | **R3** | **R4** | **R5** | **R6** | **R7** | **R8** | **R9** | **R10** | **R11** | **R12** | **R13** | **R14** | **R15** | **R16** |
| --- | --- | --- | --- | --- | --- | --- | --- | --- | --- | --- | --- | --- | --- | --- | --- |
| ABCD3  ADAM12  AP1G2  APOA2  BC002942  BLZF1  C10orf33  C14orf11  C21orf6  CDK7  DHCR24  EDNRA  FAM46C  FKBP10  FLJ10036  FLJ11336  FRAS1  IL6  KIF22  MVK  NTN4  NUP37  RARG  RNF25  RYBP  SLC4A2  SPTBN1  TAGLN  TBC1D1  WIPI49 | AGXT  ASAH1  CDK7  COL4A2  COQ6  CP  FLJ12529  FZD8  GLA  GOSR2  HT001  INSIG1  LOC221143  MAPK13  MGC11257  MOBKL1A  OSBP2  PANX1  PCOLCE  PPAP2C  PPID  PRDX2  PSMC6  PTPNS1  RABL3  RAD23A  SERPINA3  SLA  SQRDL  STK38L | C20orf19  CD68  CDC42  CLPTM1  CPSF5  DHCR24  EIF4A1  FLJ37440  H2AFX  HCFC2  HLC-8  ITGA2  LMCD1  LTBP1  MGC52110  MtFMT  NEK3  NR3C1  NSDHL  OMA1  OXSR1  PEA15  PPP1R12B  PSMC6  SLC5A10  TGFB1  THRAP1  TP53INP2  UBE2L6  WWTR1 | ADORA2B  AP1G2  APOA1  ASCC2  BFAR  BUB1B  CDC2  CLCN6  CSPG4  DC13  EIF3S10  FDFT1  FLJ12584  HELLS  ITGA2  ITM2C  JTV1  LTBP4  MPHOSPH1  NOPE  PEA15  POLR2C  PRSS23  RAB32  SARA2  SPRY2  STARD4  UBE2H  WDR37  XYLT1 | APOA1  ATF7  C14orf87  C20orf140  CAMK1  CCNH  DR1  EZH2  FKBP14  GNPNAT1  GOSR2  GRB2  HDHD2  HSU79274  IGBP1  INSIG1  KIAA1536  KLF13  KRT8  LOC56902  LOC90799  NOMO3  NRIP3  OMA1  SFRS5  STK39  TAGLN3  TCF4  eIF2A | ABI3BP  ADAMTS2  COL1A2  CXCR4  DPM2  FBXO8  FEN1  GNPNAT1  GPATC4  HNRPM  HS3ST3A1  KIF22  OSBP2  PCCB  PHB  POLR2H  PSIP1  PSMB2  PUS1  RARG  RHOBTB3  ROBO3  SFRS5  SLC22A18  SOX12  TMEPAI  TUSC3  UBQLN1  VKORC1L1  YME1L1 | BFAR  CGI-69  CP  CST3  DJ462O232  ESM1  FBXO28  FCER1G  GAS1  GPC1  HELLS  HMGCS1  IFI30  ITIH2  KDELC1  MGC52110  NUDT1  PEA15  SEMA4A  SIAT4C  SLC25A11  SMS  SRF  THRAP1  TP53INP2  UBE2L3  WEE1 | ABI3BP  AP1G2  ASCC2  BEX1  CGI-69  COL4A2  DPM1  FBXW2  FLJ12529  FLJ12875  FLJ21439  FLJ43855  FOXM1  FRAS1  HSU79274  INSIG1  ITGA6  KIAA0652  L1CAM  PAK2  PAK4  PDK2  S100A8  SFRS5  SLC16A3  SRR  TAGLN3  TUSC3  TYROBP  ZDHHC18 | ACTA2  AGPAT5  ASCC2  B3GALT6  BRD8  CNOT7  CORO7  CTSB  DOT1L  FEN1  H41  IGBP1  ILK  INPPL1  KIAA0652  LIG3  LPIN1  MXI1  MYST1  NDUFA9  PIGN  PLEKHC1  RHOBTB3  S100A11  SF3B14  TAGLN  TNRC6  URB | ASS  C14orf43  CD8B1  CDC42EP4  CNIH  CNOT7  CRABP2  CSNK1D  DDX54  DNAJB4  DUSP14  EBNA1BP2  FECH  FKBP1A  GLIS2  GPATC4  GSPT1  GTF2E2  ITGA2  ITGAL  KRT13  LOC112476  LOC51066  MYLIP  PPP4C  PRIM1  RBM5  SEC22L1  SLA  SUCLG1 | ACIN1  ARF1  ASAH1  AVEN  B3GALT6  BNC1  DPM2  EHBP1  HSPA1B  ILK  KIF22  KRT7  LOC152195  LTBP4  MIR  NOPE  NRIP3  PHB  PLK1  PLXNA1  PMP22  RABL3  SCYE1  SFRS5  SLIC1  SSR3  TCF4  THRA  VKORC1L1  XRN1 | ANXA11  BCL2L1  BLZF1  C20orf121  CAMLG  DC13  DDX49  FBXW11  FCER1G  HNRPC  HSPB8  KLF13  KRT19  LOC90799  MKKS  MYC  NUP93  PARD6A  PDK2  PHB  PMP22  PPP1CA  PUS1  RBM5  RNF25  RRM2  XYLT1  eIF2A | ACTB  C10orf33  C20orf43  CAMLG  CCR1  CGI-69  CTL2  DCK  EBNA1BP2  EDN1  EXOSC5  FLJ12584  IMPA2  JUND  KLF4  KRT8  LOC112476  MCM3  NUCB2  OMA1  PGRMC1  PLK3  PRIM2A  PRRG1  PSME3  ROBO3  TBRG4  VEGFC  VTN | ACAD9  ACTR3  APEH  C14orf1  C14orf43  C9orf77  CAMK1  CRABP2  CREB3L2  CYBB  DPM2  DUSP3  EEF1E1  FLJ20507  GREM1  H2AFX  ICMT  ITGA6  KRT19  LOC63929  MRPS33  PDLIM2  PSMD12  RAB22A  S100A8  SLC22A18  SLC7A1  TNFRSF1B  TXNDC  VBP1 | 384D8-2  AFP  ARL6IP6  C11orf13  CLN3  CRIM1  EYA3  FGFR1OP2  FGL2  GAS1  H41  HELLS  KIAA0409  KIF11  KRT19  LPIN1  MRPS35  MVK  PMP22  RKHD2  RNASEH2A  RNU3IP2  SLA  SLC25A11  STRA13  TACC3  VEGFC  XRN1  ZNF326 | ACIN1  AHCY  ARV1  ASCC2  BCL2L1  C20orf129  CANX  CDC42EP4  CICE  COX15  DHX16  DKFZP564B167  DOT1L  DR1  EHBP1  ERF  FLJ43855  GNB5  GTF2E2  KRT17  LOC51066  LOC56902  LOXL2  MGC11257  PRKRA  STC2  STRA13  TIMM17B  TNFRSF1B |
| **R17** | **R18** | **R19** | **R20** | **R21** | **R22** | **R23** | **R24** | **R25** | **R26** | **R27** | **R28** | **R29** | **R30** |  |  |
| A1BG  ATP5J  AXIN2  CARD11  CRYM  DKFZP564I0422  DKFZP564O0823  FBXO8  FLJ12448  FLJ23235  FLJ31139  FOXD4  GPR62DPCR1  HIST1H4J  HPRCACNA1H  HSD11B1  IGFBP1  MGC9850  MTX1  OTOP1  PCDHA4  PCDHB2  PRKRIR  RP9  RPLP2  SLC5A5  SOX12  TOR1B  UCP1  WFIKKNRP | ACCN4  BLOC1S3  C16orf33  C6orf190  CD3Z  CGBP  CHDC1  CLEC3B  CREB3  CSPG2  CST6  DOCK1  DUSP5  HNRPAB  HRASLS  KIAA1892  LOC51035  MGC17943  MGC3329  MRPS28  NANOS1  NMI  NTRK3  PAWR  SF3B1  SLC12A8  TES  TRPC7  VANGL2  ZCCHC12 | ASB12  ASXL1  C2orf6  C6orf31  C9orf81  CACNG4  CDC25C  CLCN1  DNAJB8  DNAPTP6  GABPB2  GTF2IRD2  HDAC2  JPX  KIAA1467  KIF3A  LCP2  LMO4  LOC143678  MGC40214  NFKBIE  NOL5A  PLEKHB1  RAB30  SCN10A  SMARCAD1  TAF9  TNKS1BP1  TRD  TSPAN16 | ACADSB  ACAT2  ACBD4  CD44  DTX2  FGD5  FLJ22169  FLJ35894  H3F3B  HOXD11  JARID1D  KIAA1706  MGC32871  OR4N4  RAB3IL1  ROBO1  RUSC2  SEMA3E  SIRT1  SLC38A3  SLC41A1  SNCG  STAC2  TAF1A  TBX19  THSD1  TM7SF2  UBE1C  ZC3H12A  ZNF593 | ABCC6  AP1S3  C16orf33  C1orf9  CCNI  CGI-14  DIRC2  DKFZp434F054  EBRP  EFS  EPHA1  FACL5  FLJ10925  FLJ21019  GFPT1  GLMN  GTPBP5  KIAA0169  LOC131368  LOC341346  MESP1  NS3TP1  PF4PF4V1PFKFB1  SAMD4  SAMSN1  SHB  TIGD2  TNFAIP6  YRDC | ACE  ALDOA  AMIGO3  ARL10C  BANF1  BSND  C10orf58  C14orf28  CBS  CD14  COX5B  CPZ  CYP11B1  DDX48  DKFZp547A023  DXS1008E  FHL3  IFNK  LOC151194  LOC92912  MBD2  MSH3  NDUFA2  PGRMC1  RBM20  RHEBL1  SLC25A10  STAB1  TSGA2  ZNFN1A3 | AFF2  AOAH  BARD1  BIN3  C11orf2  C4BPB  C9orf21  CNGB3  DDX24  FLJ10572  GDNF  GPC3  KHSRP  LALBA  LGALS3BP  LOC441500  MGC10765  MIST  MYO7A  PTGER3  QPCT  RBM21  RNF39  SETBP1  TBX21  TETRAN  TM9SF2  TXNL2  VPS35  ZDHHC5 | ATF3  C13orf7  C3orf4  C9orf4  CAPZA3  DAPP1  DDX20  DHX30  DOK4  FLJ13391  FLJ23091  FXYD2  HIC2  ITSN1  KCTD5  LOC123722  LRRC5  MMP3  MUTED  NFKBIL1  PEX5R  PPP2R2D  RBM8A  RGS2  TBCC  USP3  ZNF133  ZNF313  ZNF488 | ABL1  ASAH1  C10orf8  C6orf133  DCK  DKFZP434F091  DOCK3  FLJ22471  FLJ42486  HARS2  HSPA5BP1  LOC85865  MASP1  MBD1  MGC13379  MPP4  NTAN1  PDE7A  PLRG1  PPEF1  PQBP1  PTGER4  SH2D1A  SORCS3  SPI1  SULT4A1  TREM2  TRIM10  TRIM35  TRSP | ACVR1B  ADRA1D  ANK3  APC2  AUTL1  C21orf25  FCHSD2  FLJ31657  FLJ39249  IDUA  JRK  KENAE  LOC339448  LRCH4  NCR1  OR5B12  PCDHGA7  PLAB  PLOD  POLA  RNASEP1  RNGTT  S100A16  SELK  SLC22A12  SLC4A3  SMCHD1  TRHDE  TUBGCP5  ZNF673RHBDL2 | AP4B1  BECN1  BM-002  CASP7  COL8A1  CYGB  DPYD  EVC2  FLJ10486  FLJ20225  FLJ20287  FLJ36888  HABP2  HGRG8  HH114  HSF2BP  HSGT1  KIAA0217  OPRL1  PAK1IP1  PTCH  RAB17  SAA4  SLC6A8  SYT3  THBS4  TPSG1  UMPS  WHSC1L1  ZNF395DEFB103A | ABCA13  ACADM  ATP6V1F  C20orf32  CHRM3  COL19A1  FBXL2  FLJ22624  GAS8  HOXA1  LOC90799  MAOA  MYO18B  NICAL  NUP205  NUP50  PDF  PEX14  PPL  PRDM9  RFC4  SH3KBP1  SIGIRR  SQLE  ST3GAL2  SUV39H1  TBX22  TPSB1  TRIM65  WNK4 | AMACR  C3IP1  CXCL1  DKK2  FLJ32028  FLJ38705  FUT1  GABRP  KIAA1309  KLK11  LOC56851  MAST2  MGC1203  MTSG1  MVD  PDK2  PPAP2C  PSMD2  PTPRF  RAE1  SCN4A  SERPIND1  SLC6A13  SNX9  TAC1  TBX5  UBN1  VAMP4  ZBTB5 | AGRP  APOB48R  C16orf33  CORO1C  CYP7B1  DHRS1  DNAJC7  EFEMP1  ENTPD6  FLJ22875  KIAA1627  KPTN  MGC13017  MGC15716  MGC17943  NYD-SP26  OTUB1  PRKAB2  PRSS7  RGS9BP  RNPEP  RPS7  SNAP29  SND1  SV2C  TEX15  TNFSF6  WDR69ZSWIM2  ZC3HDC3 |  |  |

**Additional file 3. Randomly selected gene groups.**
