# Supplementary material for: Analysis of promoter regions of co-expressed genes identified by microarray analysis
Source: BMC Bioinformatics. 2006 Aug 17;7:384. doi: 10.1186/1471-2105-7-384 (PMC1560170; doi:10.1186/1471-2105-7-384)
Supplement: Additional File 4 — Promoter analysis for the randomly selected gene groups. [file 1471-2105-7-384-S4.doc]

Additional file 4. Promoter analysis for the randomly selected gene groups.

| **Clusters** | **Subclusters** | Significant TFBS present in all promoters of a given cluster |
| --- | --- | --- |
| 1 | 1A | No Significant |
|  | 1B | Significant TFBS but not present in all (GATA1, IPF1, NFKB) |
|  | 1C | Significant TFBS but not present in all (E2F, NFKB) |
|  |  |  |
| 2 | 2A | No Significant |
|  | 2B | Significant TFBS but not present in all (E2F, HTF) |
|  |  |  |
| 3 | 3A | No Significant |
|  | 3B | Significant TFBS but not present in all (AHR, E2F, GATA1, IK1, LPOLYA, NFKB, NFY, TGIF, USF, YY1) |
|  |  |  |
| 4 | 4A | CAAT, CETS168, CETS1P54, CREL, HAND1E47, IK1, LDSPOLYA, MAZ,MYOD, RFX1, TCF1P |
|  | 4B | Significant TFBS but not present in all (E2F) |
|  |  |  |
| 5 | 5A | Significant TFBS but not present in all (E2F) |
|  | 5B | Significant TFBS but not present in all (ARNT, E2F, GATA1, GATA2, HIF1, TATA) |
|  |  |  |
| 6 | 6A | No Significant |
|  | 6B | No Significant |
|  | 6C | Significant TFBS but not present in all (E2F, AHR) |
|  |  |  |
| 7 | 7A | Significant TFBS but not present in all (E2F, NFKB) |
|  | 7B | Significant TFBS but not present in all (CETS168, ELK1, GABP, IPF1, NRF2) |
|  |  |  |
| 8 | 8A | Significant TFBS but not present in all (AHR, ATF, CREB, E2F, E4F1, HIF1, HTF, XBP1) |
|  | 8B | Significant TFBS but not present in all (CREL, E2F, E2F1, USF) |
|  |  |  |
| 9 | 9A | No Significant |
|  | 9B | Significant TFBS but not present in all (SRF, AHR) |
|  |  |  |
| 10 | 10A | GATA3 |
|  | 10B | No Significant |
|  |  |  |
| 11 | 11A | CAAT, CEBPDELTA, FOXO4, HFH8, IK1 |
|  | 11B | No Significant |
|  | 11C | Significant TFBS but not present in all (HIF1, ARNT) |
|  |  |  |
| 12 | 12A | CAAT, ETS1, NFY |
|  | 12B | Significant TFBS but not present in all (E2F1DP1, HNF1, NFY) |
|  | 12C | Significant TFBS but not present in all (E2F) |
|  |  |  |
| 13 | 13A | No Significant |
|  | 13B | Significant TFBS but not present in all (E2F) |
|  |  |  |
| 14 | 14A | ER, FOXO4, GATA1, HNF3ALPHA, IK1, RORA1 |
|  | 14B | Significant TFBS but not present in all (E2F, HNF4) |
|  |  |  |
| 15 | 15A | Significant TFBS but not present in all (E2F) |
|  | 15B | Significant TFBS but not present in all (SRF) |
|  |  |  |
| 16 | 16A | Significant TFBS but not present in all (AHR, OLF1) |
|  | 16B | No Significant |
|  | 16C | Significant TFBS but not present in all (AHR, CREBP1CJUN) |
|  |  |  |
| 17 | 17A | No Significant |
|  | 17B | No Significant |
|  |  |  |
| 18 | 18A | **CACCCBINDINGFACTOR, CETS168, CMYB, E2F1, ER, ETS1, ETS2, GABP, IK1, IK3, LDSPOLYA, MAZ, MYOD, NFKB, NRF2, RORA1, TFIIA** |
|  | 18B | No Significant |
|  |  |  |
| 19 | 19A | No Significant |
|  | 19B | Significant TFBS but not present in all (AHR, CETS168, CREL, ETS2, NFKAPPAB, NFKB, NKX61, TCF1P) |
|  |  |  |
| 20 | 20A | Significant TFBS but not present in all (EVI1, HIF1, SREBP1, ZID) |
|  | 20B | Significant TFBS but not present in all (HNF4, MYOD, S8) |
|  |  |  |
| 21 | 21A | No Significant |
|  | 21B | Significant TFBS but not present in all (AHR, IPF1, MYOD, TAL1BETAE47) |
|  |  |  |
| 22 | 22A | No Significant |
|  | 22B | No Significant |
|  |  |  |
| 23 | 23A | Significant TFBS but not present in all (FREAC7, GATA1, GATA2, HNF1, HNF4, LMO2COM, LPOLYA, NFY, NRF2, S8, TAL1BETAITF2, TATA, XFD2) |
|  | 23B | Significant TFBS but not present in all (HNF1) |
|  |  |  |
| 24 | 24A | Significant TFBS but not present in all (NFKB) |
|  | 24B | CEBPDELTA, ETS1, GATA, GATA1, LMO2COM, MYOD |
|  |  |  |
| 25 | 25A | No Significant |
|  | 25B | No Significant |
|  |  |  |
|  |  |  |
| 26 | 26A | GATA1, SOX5 |
|  | 26B | No Significant |
|  |  |  |
| 27 | 27A | Significant TFBS but not present in all (NFKB, STAT, YY1) |
|  | 27B | No Significant |
|  |  |  |
| 28 | 28A | Significant TFBS but not present in all (GATA2, MMEF2, MYCMAX, NFY) |
|  | 28B | Significant TFBS but not present in all (E2) |
|  |  |  |
| 29 | 29A | No Significant |
|  | 29B | Significant TFBS but not present in all (FOXJ2, MEF2, NFKB) |
|  |  |  |
| 30 | 30A | Significant TFBS but not present in all (IPF1) |
|  | 30B | No Significant |
|  |  |  |
