# Supplementary material for: Analysis of promoter regions of co-expressed genes identified by microarray analysis
Source: BMC Bioinformatics. 2006 Aug 17;7:384. doi: 10.1186/1471-2105-7-384 (PMC1560170; doi:10.1186/1471-2105-7-384)
Supplement: Additional File 5 — Gene members in the AML PTM clusters. [file 1471-2105-7-384-S5.doc]

Additional file 5. Gene members in the AML PTM clusters^1^.

| Cluster 1 | Cluster 2 | Cluster 3 | Cluster 4 | Cluster 5 | Cluster 6 | Cluster 7 | Cluster 8 | Cluster 9 | Cluster 10 |
| --- | --- | --- | --- | --- | --- | --- | --- | --- | --- |
| APN2 ABCF1  ANXA2  ARHGEF11  C19orf6  CD68  CST3  CTNNA1  CTSB  CTSH  CYBB  CYP51A1  FCER1G  HEXB  IFI30  IL13RA1  KIF1C  LCP1  LOC152195  MAP2K1  MSP  PTPNS1  RAD23A  RER1  RIN1  S100A11  S100A4  TIMP1  ZNF451 | E2-230K ALS2CR3  BCL2L1  BPGM  C14orf87  CGI-69  EPB41  FECH  GSPT1  GSPT2  HAGH  HRI  HT014  MIR  MSCP  PRDX2  PRKWNK1  SELENBP1  TM4SF9  WDR40A | FOXO3A ADIPOR1  BNIP3L  CPEB4  CSNK1D  HIF1A  MAGEA10  MAX  MXI1  RIOK3  RNF10  RNF159  SPTBN2  UBE2H | GLIS2 ARFRP1  C19orf24  CDK10  EIF4EBP1  FBXL7  MAD1L1  Magmas  MRPL55  NDUFB7  PHKG2  POLR2H  SEMA5A  TETRAN | MAX C1orf16  C20orf140  CDC42  CHD2  CLIC4  CSNK1D  DKFZp566C0424  DNAJC3  FAM53C  FLJ30656  FOXO3A  FXR1  IL1RAP  KIAA0033  KIAA0143  KIAA0652  KIAA0692  LOC168850  MAGEA10  MAP3K7IP2  MGC4268  MLL3  MYST2  NFAT5  PHF5A  PIK3CA  PPP4R1  RAB6A  RIOK3  RKHD2  RNF10  SLC38A2  SP3  SPTBN1  SRPR  STAT3  SYAP1  TGOLN2  UBE2H  YAP  ZFR  ZNF326  ZNF395  ZNF644 | MYBL2 ANLN  BIRC5  BRRN1  BUB1B  C18orf24  CDC6  CDCA1  CDCA8  CDT1  DLG7  FEN1  H2AFX  HMMR  HSPC150  KIAA0101  LOC388962  MELK  MLF1IP  PLK1  POLE2  PRIM1  PTTG1  RECQL4  TK1  TPX2  UBE2C  ZWINT | SP3 ABHD2  C1orf16  C20orf140  CD44  CLIC4  CNOT6L  CSNK1D  DKFZp566C0424  DNAJC3  DVL3  ERBB2IP  FAM53C  FLJ12529  FLJ20259  HAPIP  KIAA0143  KIAA0652  KIAA0692  KIAA1109  LAPTM4A  LOC90799  MAP3K1  MAP3K7IP2  MAX  MFAP3  MGC4796  MLL3  MOBK1B  MYST2  PCTK2  PRO0149  PRPF8  RAB6A  RKHD2  SEC24A  SLC38A2  SSR1  STAT3  TAB3  TGOLN2  TMEM33  TMP21  TNRC6  ZCCHC6  ZFR  ZNF395  ZNF644 | STAT3 ATXN7  CSNK1D  DKFZp566C0424  EIF4G1  KIAA0143  KIAA0652  MAP3K7IP2  MAX  PHF5A  PIK3R1  PPP4R1  PTPN1  RAB6A  SHC1  SP3  YAP  ZFR | TGIF2 C1orf16  CNOT4  DDX42  KIAA1586  KPNA5  MLL  PIK3R1  PPP4R1  RBM5  SMARCC1  SPTBN1  TCERG1  TNRC6  USHBP1  ZFR  ZNF326 | VDR APOBEC3B  CCR1  CD14  CD68  CSPG2  CYP1B1  CYP51A1  DTX4  IFI30  ITGB2  KRT5  LIG4  LILRB2  MSP  PIK3R5  PRRG1  PTAFR  PTPNS1  SLA  TNFRSF1B  UNC119 |

^1^The DNA binding transcription factor encoding genes that their binding sites information present in TRANSFAC database are colored in red.
